# Single inhalation exposure to polyamide micro and nanoplastic particles impairs vascular dilation without generating pulmonary inflammation in virgin female Sprague Dawley rats

**DOI:** 10.1186/s12989-023-00525-x

**Published:** 2023-04-23

**Authors:** Chelsea M Cary, Talia N Seymore, Dilpreet Singh, Kinal N Vayas, Michael J Goedken, Samantha Adams, Marianne Polunas, Vasanthi R Sunil, Debra L Laskin, Philip Demokritou, Phoebe A Stapleton

**Affiliations:** 1 Department of Pharmacology and Toxicology Ernest Mario School of Pharmacy, Environmental and Occupational Health Sciences Institute, Rutgers University, 170 Frelinghuysen Road, 08854 Piscataway, NJ USA; 2 Center for Nanotechnology and Nanotoxicology, Department of Environmental Health, Harvard T. H. Chan School of Public Health, Harvard University, 02115 Boston, MA USA; 3 Research Pathology Services, Rutgers University, 08854 Piscataway, NJ USA; 4 Environmental and Occupational Health Sciences Institute (EOHSI), 08854 Piscataway, NJ USA; 5 Department of Environmental and Occupational Health and Justice, Rutgers School of Public Health, Rutgers University, 08854 Piscataway, NJ USA

**Keywords:** Microplastic, Nanoplastic, Inhalation, Particle, Polyamide, Nylon, Cardiovascular, Inflammation, Endocrine disruption

## Abstract

**Background:**

Exposure to micro- and nanoplastic particles (MNPs) in humans is being identified in both the indoor and outdoor environment. Detection of these materials in the air has made inhalation exposure to MNPs a major cause for concern. One type of plastic polymer found in indoor and outdoor settings is polyamide, often referred to as nylon. Inhalation of combustion-derived, metallic, and carbonaceous aerosols generate pulmonary inflammation, cardiovascular dysfunction, and systemic inflammation. Additionally, due to the additives present in plastics, MNPs may act as endocrine disruptors. Currently there is limited knowledge on potential health effects caused by polyamide or general MNP inhalation.

**Objective:**

The purpose of this study is to assess the toxicological consequences of a single inhalation exposure of female rats to polyamide MNP during estrus by means of aerosolization of MNP.

**Methods:**

Bulk polyamide powder (i.e., nylon) served as a representative MNP. Polyamide aerosolization was characterized using particle sizers, cascade impactors, and aerosol samplers. Multiple-Path Particle Dosimetry (MPPD) modeling was used to evaluate pulmonary deposition of MNPs. Pulmonary inflammation was assessed by bronchoalveolar lavage (BAL) cell content and H&E-stained tissue sections. Mean arterial pressure (MAP), wire myography of the aorta and uterine artery, and pressure myography of the radial artery was used to assess cardiovascular function. Systemic inflammation and endocrine disruption were quantified by measurement of proinflammatory cytokines and reproductive hormones.

**Results:**

Our aerosolization exposure platform was found to generate particles within the micro- and nano-size ranges (thereby constituting MNPs). Inhaled particles were predicted to deposit in all regions of the lung; no overt pulmonary inflammation was observed. Conversely, increased blood pressure and impaired dilation in the uterine vasculature was noted while aortic vascular reactivity was unaffected. Inhalation of MNPs resulted in systemic inflammation as measured by increased plasma levels of IL-6. Decreased levels of 17β-estradiol were also observed suggesting that MNPs have endocrine disrupting activity.

**Conclusions:**

These data demonstrate aerosolization of MNPs in our inhalation exposure platform. Inhaled MNP aerosols were found to alter inflammatory, cardiovascular, and endocrine activity. These novel findings will contribute to a better understanding of inhaled plastic particle toxicity.

## Background

Human exposure to micro- and nanoplastic particles (MNPs) has gained significant attention in the past few years due to their increased production, widespread distribution in the environment, and recent detection in human tissues and fluids [1–6]. MNPs are generated through mechanical, thermal, and photochemical degradation of the millions of tons of plastic produced and discarded each year [7]. The resulting sizes of the particles generated by these processes fall in the micro- (< 5 mm and > 100 nm) or nano- (< 100 nm) ranges allowing the fraction of particles that are 10 μm or less to be readily aerosolized and inhaled. The potential for ubiquitous exposure, combined with the current knowledge of the toxicity of aerosolized particles, raises questions about the health effects of MNP inhalation.

Recent studies have detected airborne MNPs in both indoor and outdoor environments [8–10] and unequivocally identified microplastic particles in lung samples from patients and human cadavers [3, 5]. Occupational inhalation of plastic nylon fibers has been shown to cause pulmonary inflammation known as Flock disease or Flock worker’s lung [11]. Earlier studies have demonstrated that aerosolized particles like particulate matter (PM) or engineered nanomaterials (ENM) cause aberrant pulmonary function [12–14], pulmonary inflammation [14–20], systemic inflammation [17, 21–25], and impaired cardiovascular activity [15, 17, 23–30].

A variety of adverse cardiovascular outcomes can occur after aerosolized particle exposure. Affected portions of the cardiovascular system include the heart [31–33], the macrocirculation (conduit vessels > 150 microns in diameter) [34, 35], and the microcirculation (vessels < 150 microns) [36–39]. Exposure to particulate mixtures such as PM or diesel exhaust often lead to increases in blood pressure [40, 41], whereas ENM exposure do not generate these changes [42, 43]. Vascular dysfunction has also been described after particulate exposure [42, 44, 45]. This is characterized by decreased vasomotor function of the vessels in response to endothelial or vascular smooth muscle stimuli. Effects on the macrocirculation and microcirculation, however, are often different, and may vary between vascular beds [43, 46–48]. Changes in cardiovascular function after particle inhalation are thought to occur as a consequence of systemic inflammation, direct particle interaction with vessels, and/or decreased neurological control of the heart and vasculature [49]. The effect of MNP inhalation on the cardiovascular system are unknown.

The inflammatory and cardiovascular effects of particle inhalation vary depending on physicochemical properties including particle chemistry, size, and surface area. Among MNPs, different plastic polymers exhibit unique physicochemical properties that influence their interactions with biological tissues. Plastics are a toxicologically unique aerosol because polymer materials are frequently manipulated with chemical additives, known as plasticizing compounds, often to promote flexibility or hardness characteristics in the final products. These chemical additives, including bisphenols and phthalates, have been identified as endocrine disruptors due to their ability to mimic endogenous reproductive hormones [50]. Disruption of reproductive hormones, including estradiol and progesterone, may also play an important role in vascular responsivity and systemic inflammation [51, 52]. While many studies have theorized that MNPs may act as a vector for endocrine disruption chemicals, these outcomes remain unclear.

The purpose of this study was to characterize MNP aerosol toxicity in a whole-body rodent inhalation exposure model utilizing a novel MNP. Polyamide served as a representative plastic test material that is not produced with bisphenol analogs or phthalates [53–55] and has no known endocrine disrupting properties. We observed robust cardiovascular alterations and systemic inflammatory and endocrine responses following polyamide exposure of female rats during estrus. These results are important as they are the first to demonstrate whole-body polyamide MNP inhalation in a laboratory model for the assessment of systemic toxicities. Furthermore, we identified toxicities pertaining to inflammatory, cardiovascular, and endocrine activity.

## Results

### Particle characterization

**Fig. 1:**
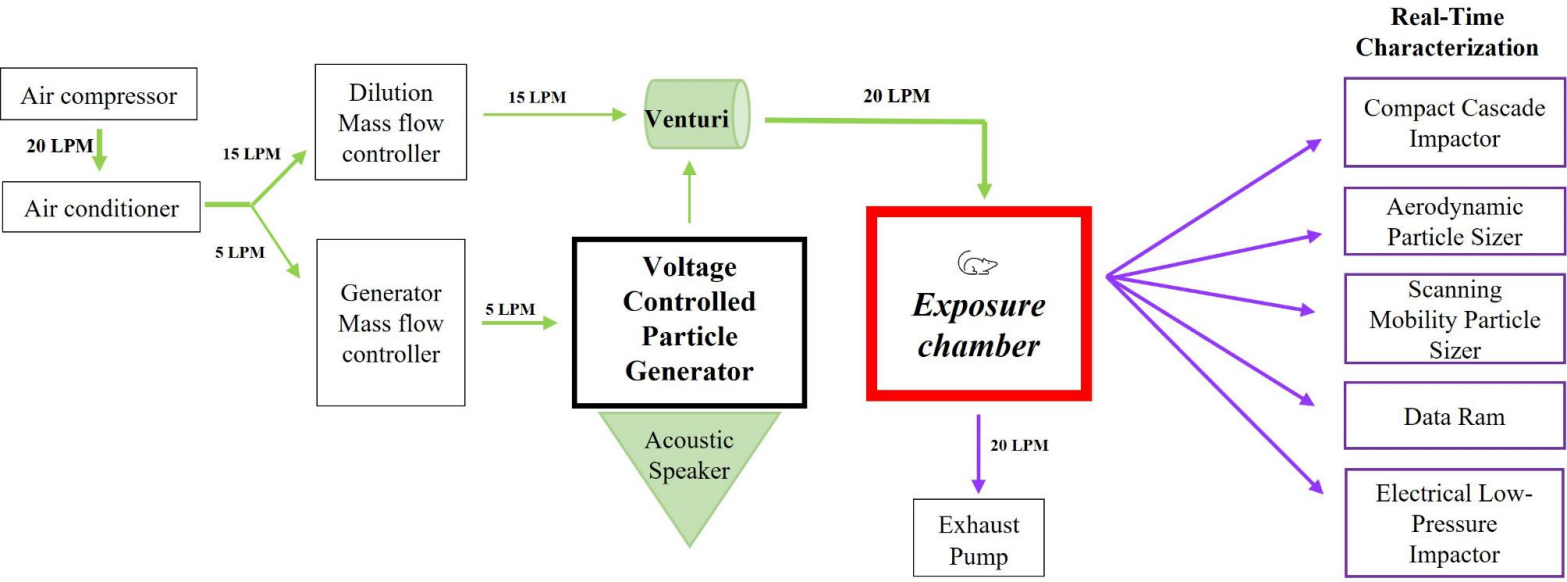
General schematic of the rodent whole-body inhalation platform used for experimentation

**Fig. 2:**
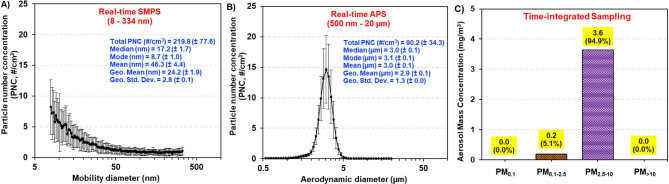
Real-time size characterization of the whole-body inhalation facility shows the presence of nanoscale particles (**A**), and microparticles (**B**). Time-integrated characterization of the overall particle size fractionation during exposure was quantified using a Harvard Compact Cascade Impactor (**C**). Data are presented as mean ± SEM.

The measured Brunauer–Emmett–Teller (BET) specific surface area (SSA) of the polyamide powder was 9.89 ± 0.55 m^2^/g and the calculated equivalent BET diameter of the particles was 0.53 ± 0.03 μm. A visual representation of the whole-body inhalation platform used for polyamide powder aerosolization is shown in Fig. 1. The total aerosol number concentration measured by the Scanning Mobility Particle Sizer (SMPS) (8–300 nm) averaged 220 ± 78 particles/cm^3^ (range: 110–356 particles/cm^3^) with a count median mobility diameter of 17.2 ± 1.7 nm (Fig. 2A). The average particle number concentration from the Aerodynamic Particle Sizer (APS) (0.5–20 μm) was 90 ± 34 particles/cm^3^ (range: 42–334 particles/cm^3^) with a narrow unimodal size distribution having a median aerodynamic diameter of 3.0 ± 0.1 μm (Fig. 2B). The bulk of the PM mass (~ 95%) was contained within the PM_2.5−10_ aerodynamic size fraction and the remaining mass (~ 5%) resided in the PM_0.1−2.5_ range as measured by the Harvard Compact Cascade Impactor (CCI) (Fig. 2C). This is in agreement with APS data and was confirmed using a High-Resolution Electrical Low Pressure Impactor (HR-ELPI) (data not shown). These data identify the polyamide aerosols as MNPs with a median diameter of 2.81 μm (GSD = 1.3). As the aerosolization platform produced respirable particles throughout the exposure, the time-weighted average for which rats were exposed is lower than OSHA’s permissible exposure limit for particles of this size. Characterization of the filtered air exposure revealed negligible levels of particles produced by hair, dander, and excrement (data not shown).

### Animal characteristics and pulmonary deposition modeling

Animal age and heart weight were not significantly different between the control and exposure groups. Inhalation of polyamide MNP resulted in a 14% increase in mean arterial pressure (MAP) (Table 1).

**Table 1:** Animal age and weight, heart weight, and mean arterial pressure. Data are presented as mean ± SEM, n = 9–17, *Significantly different (p < 0.05) from control animals as determined by a two-tailed t-test assuming equal variance between groups

Characteristic	Age (weeks)	Body Weight (g)	Heart Weight (g)	Mean Arterial Pressure
Control	11.6 ± 1.3	211 ± 8	0.67 ± 0.03	74.3 ± 2.7
Polyamide	13.6 ± 1.0	233 ± 5*	0.72 ± 0.02	85.2 ± 2.7*
Polyamide (Bronchoalveolar lavage subset)	10.7 ± 0.3	226 ± 3	N/A	N/A

Without clearance, the estimated mass fraction of the inhaled particles that was deposited across the different respiratory regions was 76.7%, with the most deposition predicted in the head (extra-thoracic) region (49.9%), followed by the tracheobronchial (23.4%), and alveolar (3.4%) regions. The predicted deposited particle doses in these regions over the 4 h exposure period were 21.8, 3.25, and 0.035 µg/cm^2^, respectively. Assuming particle clearance for 20 h after the end of the exposure period, the estimated retained doses in the tracheobronchial and alveolar regions were 0.003 and 0.033 µg/cm^2^, respectively, indicating a high rate (> 99.9%) of clearance from the tracheobronchial area and negligible clearance from the alveolar region. No clearance calculation for the head region was available in the Multiple-Path Particle Dosimetry (MPPD) model.

### Pulmonary histopathology and inflammation

**Fig. 3:**
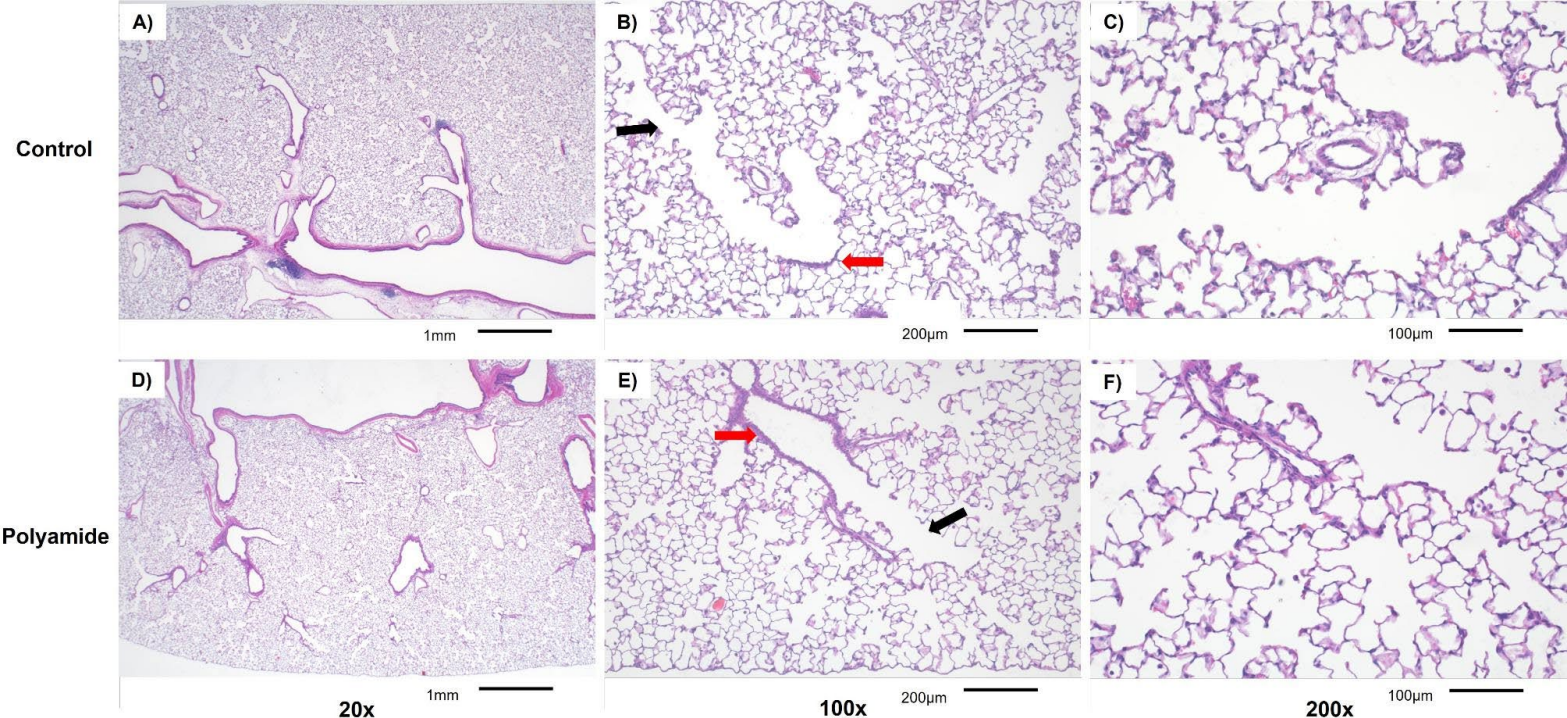
Representative images of H&E-stained sections at 20x, 100x, and 200x magnification from naïve female rat controls (n = 9) or polyamide exposed rats (n = 8) are above. Low magnification morphological appearance of the conducting airways can be seen at 20x magnification (**A** and **D**). The proximal bronchioles (red arrows) and the distal bronchioles (black arrows) are shown at a 100x magnification (**B** and **E**). Representative images of the alveolar region are shown at 200x magnification (**C** and **F**)

Histopathological analysis of pulmonary tissue did not reveal any significant differences in infiltrating immune cell populations, morphological damage to pulmonary structures, or damage to the pulmonary vasculature (Fig. 3). Pulmonary tissue showed a continuous layer of ciliated epithelia in the higher-level conducting airways in both groups (Fig. 3A-B and D-E). There were no changes in the structure of the vasculature, or thickness of parenchymal cells lining the bronchi and terminal bronchioles (Fig. 3B and E). A comparable number of macrophages and neutrophils in the alveolar sacs and vasculature was observed in both groups (Fig. 3C F). Exposure did not disrupt the continuity and thickness of the alveolar barrier or proximity to pulmonary capillaries (Fig. 3C F). Overall, the epithelial barrier remained intact and pulmonary inflammation was not identified 24 h after a single exposure to MNP aerosols in virgin female rats.

Analysis of bronchoalveolar lavage (BAL) fluid showed a significant yet physiologically negligible increase in the percentage of neutrophils in exposed animals (Table 2). No differences in protein levels or cell numbers were detected (Table 2).

**Table 2:** BAL fluid immune cell and protein levels. Significance was assessed by comparing outcomes with a two-tailed t-test assuming equal variance between groups. If data were not normally distributed and variance was unequal between groups, the Wilcoxon Rank Sum test was used for comparison of data sets. Data are presented as mean ± SEM, n = 9, *Significantly different (p < 0.05) from naive controls as determined by visual identification and quantification of Giemsa-stained cells

Percentage				Protein Concentration (µg/mL)
	Macrophage	Neutrophil	Lymphocyte	
Control	97.6 ± 1.08	0 ± 0	2.44 ± 1.09	177 ± 36.9
Exposed	97.3 ± 1.48	0.33 ± 0.13*	2.37 ± 1.54	220 ± 33.6

### Macrocirculation vascular reactivity

**Fig. 4:**
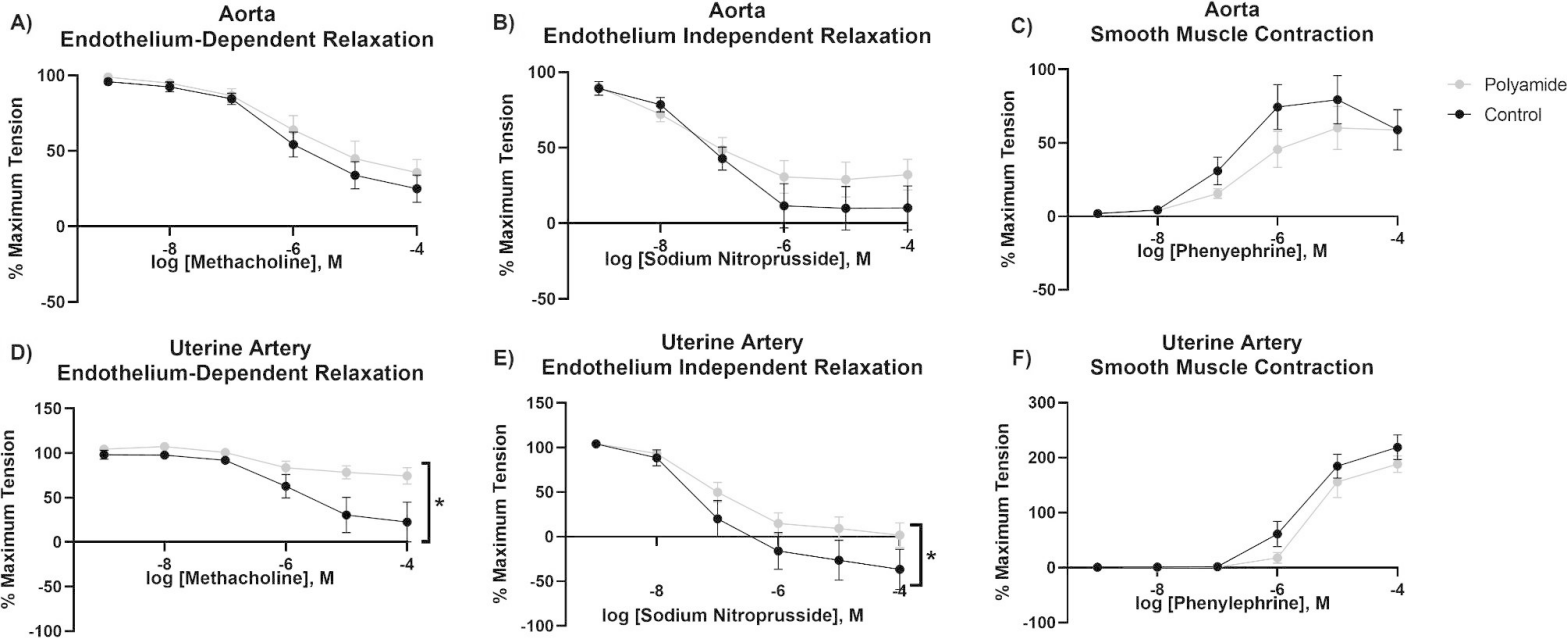
Vascular reactivity in the abdominal aorta and uterine artery was assessed by establishing concentration response curves with the endothelial-dependent vasodilator methacholine (**A** and **D**), the endothelial-independent vasodilator sodium nitroprusside (**B** and **E**), and the vasoconstrictor phenylephrine (**C** and **F**). Significance was assessed by comparing overall reactivity via a four-parameter nonlinear regression analysis. Data are presented as mean ± SEM, n = 9–11, *Significantly different (p < 0.05) from filtered air controls

Aortic and uterine artery segments were assessed for their vascular reactivity to investigate alterations within the macrocirculation (conduit vessels > 150 microns) induced by polyamide inhalation. Uterine vessels were selected for investigation to represent a distal vascular bed for which vascular reactivity varies based on estrous stage [37]. Four parameter logistic regression modeling of the concentration response curves identified no significant differences in the reactivity of the aortas in rats exposed to polyamide relative to filtered air control rats (Fig. 4A-C). However, the left uterine artery exhibited both endothelium-dependent and -independent impairment in the rate of relaxation after methacholine (MCh) and sodium nitroprusside (SNP) stimulation, respectively (Fig. 4D and E). There were no alterations in vascular smooth muscle responses after stimulation with phenylephrine (PE) (Fig. 4F). These data suggest vasodilation pathways within the macrocirculation are selectively impaired 24 h after a single exposure to MNP aerosols, while vasoconstriction pathways are spared.

### Microcirculation vascular reactivity

**Fig. 5:**
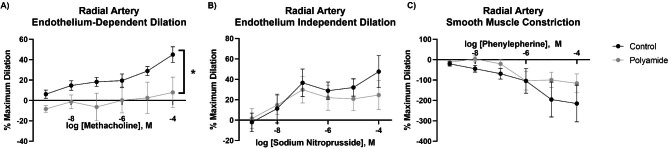
Vascular reactivity in the radial artery was assessed by establishing drug response curves with the endothelial-dependent vasodilator, methacholine (**A**), the endothelial independent-vasodilator, sodium nitroprusside (**B**), and the vasoconstrictor, phenylephrine (**C**). Significance was assessed by comparing overall reactivity via a four-parameter nonlinear regression analysis. Data are presented as mean ± SEM, n = 7–9, *Significantly different (p < 0.05) from filtered air controls

Resistance vessels are the major regulators of tissue perfusion. We assessed the vascular reactivity of the premyometrial radial artery, as a representative vessel of the microcirculation. Polyamide MNP inhalation led to a significantly decreased dilation response to MCh, an endothelium-dependent dilator (Fig. 5A). Radial artery segments from filtered air controls and MNP exposed rats exhibited comparable vascular reactivity for the endothelium-independent vasodilation and smooth muscle constriction pathways in response to SNP and PE, respectively. (Fig. 5B and C). These data indicate that MNP inhalation may impair dilation of the microcirculation primarily by affecting the endothelium.

### Systemic inflammation

**Fig. 6:**
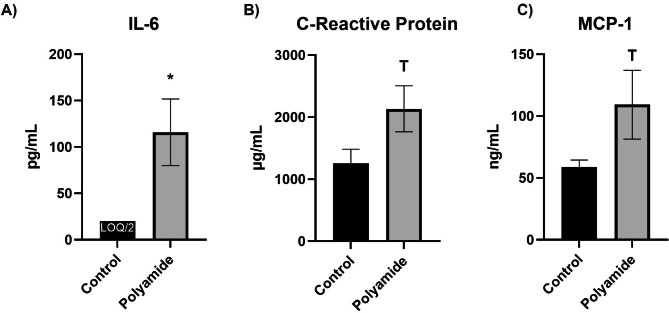
Enzyme-linked immunosorbent assays for IL-6 (**A**), CRP (**B**), and MCP-1 (**C**) were carried out for assessment of systemic inflammation. Samples with analyte levels below the level of quantification and above the level of detection were assigned the value LOQ/2. Significance was assessed by comparing outcomes with a two-tailed t-test assuming equal variance between groups. Data are presented as mean ± SEM, n = 7–9, *Significantly different (p < 0.05) from filtered air controls; ^T^Trending difference (p ≤ 0.10) from filtered air controls

Biomarkers of systemic inflammation were measured in plasma from control rats and MNP exposed rats (Fig. 6). Circulating levels of IL-6 were significantly elevated in 24 h post-MNP inhalation. CRP and MCP-1 levels were also elevated, but these values did not reach significance (p = 0.06 and 0.10, respectively).

### Hormonal mediators of vascular reactivity

**Fig. 7:**
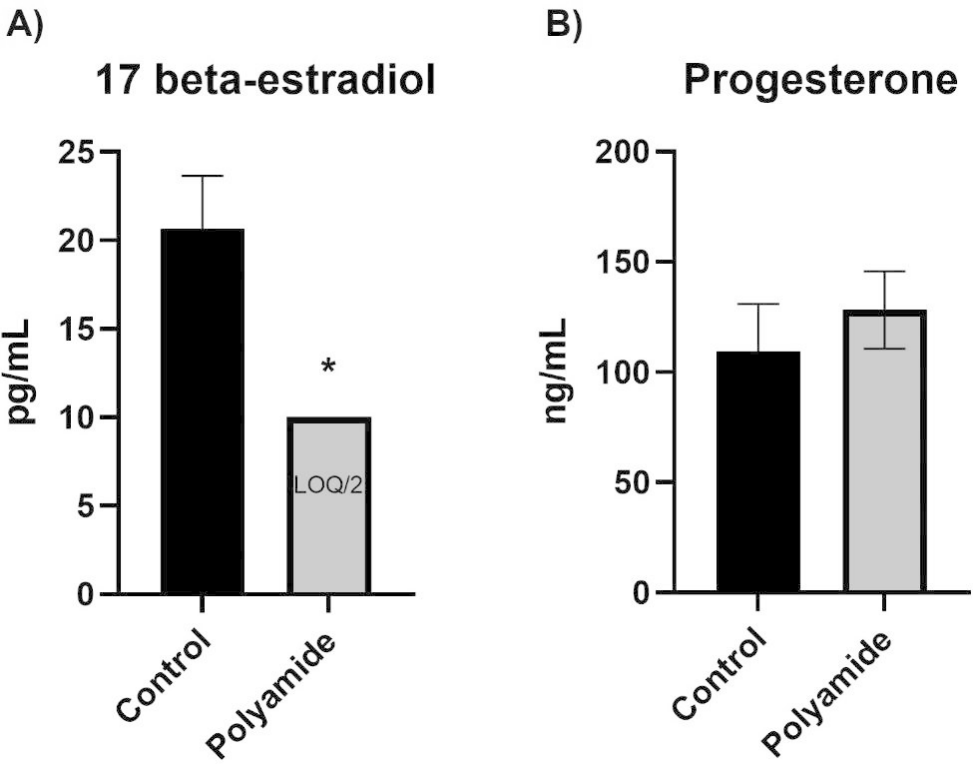
Enzyme-linked immunosorbent assays for 17β-estradiol (**A**) and progesterone (**B**). Samples with analyte levels below the level of quantification and above the level of detection were assigned the value LOQ/2. Significance was assessed by comparing outcomes with a two-tailed t-test assuming equal variance between groups. Data are presented as mean ± SEM, n = 7–9, *Significantly different (p < 0.05) from filtered air controls

In further studies, female rats were exposed during estrus to control for hormonal differences that may affect vascular reactivity. As potential endocrine disruption after MNP inhalation has not been investigated, we measured circulating levels of 17β-estradiol and progesterone in rats exposed to MNPs. Levels of circulating 17β-estradiol were significantly decreased in MNP exposed rats when compared to controls while progesterone was unaffected (Fig. 7A and B).

## Discussion

Herein, we present novel data on the pathophysiological effects of inhalation exposure of estrus-staged virgin Sprague Dawley rats to a single dose of polyamide MNPs. Our findings identify multiple adverse cardiovascular outcomes including elevated blood pressure and impaired uterine vascular dilation. MNP inhalation also induced systemic inflammation, as evidenced by increased levels of proinflammatory cytokines in plasma. Decreases in circulating levels of 17β-estradiol after MNP inhalation suggest that polyamide may be endocrine disrupting, a property that would further impact cardiovascular function.

In these studies, we characterized the inhalation exposure platform and demonstrated that polyamide MNPs can readily be aerosolized for inhalation delivery to rodents. Using time-integrated sampling, we showed that particles in the PM_2.5−10_ aerodynamic size fraction contributed most to the mass of aerosolized particles. However, the PM_< 0.1_ size fraction had the highest detectable particle number/cm^3^. In our MPPD model, the percentage of deposited mass in the alveolar region was low in comparison to the tracheobronchial region. Nevertheless, the retained dose in the alveolar region was an order of magnitude greater than the tracheobronchial region. Measurements of other solid aerosols yield comparable results in terms of particle contribution to mass [56] and pulmonary deposition [57], but differed in terms the percentage of particle mass in each size fraction [58]. It should be noted that the MNPs in this study are a pristine, bulk polyamide material, as they have not undergone significant weathering since initial production. The exposure concentration used in this study cannot yet be compared to real-world exposure assessments due to limitations in detection of nano-sized particles. The spheroidal shape of the particles used in this study differs from the fragments and fibers detected environmentally and in tissue. Physicochemical properties of secondary particles resulting from mechanical degradation or incineration may modulate the toxicological outcomes compared to what we observed in this model. Due to the lack of available research in this area, it is unclear whether the overall mobility of particles and pulmonary deposition that we observed are consistent with environmental sources of MNPs.

A single exposure to polyamide aerosol did not generate notable pulmonary inflammation in our experimental model. Thus, 24 h post-exposure, there was no apparent histopathology or inflammatory changes in lung tissue. Negligible changes in the inflammatory cell population percentages and protein levels were noted in BAL fluid. While inflammation is often associated with pulmonary particle exposure, the absence of this outcome has been described in other studies of solid aerosols, despite higher airborne particle numbers than observed in our platform [39, 59]. Whether repeated inhalation exposure to MNPs would impact pulmonary inflammation remains to be determined.

Both systemic and local cardiovascular impairments were identified in our experimental rats. Increases in MAP after MNP inhalation indicates that this exposure generates systemic vascular impairments. This observation is distinct from other classes of homogenous particles such as metallic or carbonaceous materials [42, 43]. However, exposure to 3D printing aerosols, a conglomerate of particles and carcinogenic gases [60], has been shown to elevate MAP within 24 h of inhalation exposure [38]. In our study, large conduit vessels in exposed rats showed varied changes after polyamide MNP inhalation with the aorta being unaffected while the uterine artery exhibited blunted endothelium-dependent and -independent dysfunction. Polyamide exposure generated differential outcomes in the uterine microcirculation with only endothelium-dependent dilation being impaired. Other solid aerosols impair systemic dilation after pulmonary exposure, and the impact can vary between the macrocirculation and microcirculation even when the test material is the same [44]. The uterine vasculature has been identified as a vascular bed affected by particle inhalation through varying mechanisms [37, 45, 47, 61–63]. In sexually mature female animals, the uterine vasculature must have the appropriate response to stimuli in order to support reproductive functions of uterine tissue [51, 64, 65]. Findings presented here indicate that polyamide MNPs may impact vascular beds, conduit vessels, and resistance vessels through different, as of yet unidentified mechanisms.

Our data suggest that MNP inhalation increases circulating levels of proinflammatory cytokines. Other markers of acute inflammation such as IL-4, IL-5, and IFNγ were included in our initial assessment, but these cytokines were below the limit of detection in our assays. Selection of these cytokines for measurement of systemic inflammation was based on frequency of a detectable change in these biomarkers at a 24 h post-exposure timepoint in previous studies [66–70]. IL-1β and TNFα were not included due to their earlier peak following exposure to compounds known to cause inflammation [66, 67, 69, 71]. We found that circulating IL-6 was significantly higher in MNP exposed rats 24 h after exposure; moreover, biomarkers induced by IL-6, such as CRP and MCP-1, were also elevated, however, these data were not significant. These findings are consistent with epidemiological and animal studies that suggest pulmonary particle exposure generates systemic inflammation [21–23, 69, 72–74]. Further analysis of systemic inflammation caused by MNPs is required to assess the temporal relationship between this exposure and inflammatory cytokines.

To our knowledge, this report is the first to demonstrate endocrine disruption after MNP inhalation. We selectively focused on reproductive hormones that influence vascular reactivity. We controlled for hormonal cycling by exposing female rats only in estrus, the most fertile stage of their reproductive cycle. Consequently, the observed decrease in 17β-estradiol can be attributed to the inhalation of polyamide. Recently, Dusza et al. reported that *hsd17b1*, a gene which regulates 17β-estradiol biosynthesis, was downregulated after MNP exposure in a placental cell culture model [75]. This outcome occurred independently of systemic inflammation. To our knowledge, there is no evidence to suggest that systemic inflammation modulates reproductive hormone production unless the inflammation is severe enough to affect hormone producing tissues. However there is evidence indicating that estrogen and progesterone may act as anti-inflammatory compounds or in the resolution of inflammation [76–79]; therefore, it is more likely that the reduction in 17β-estradiol reported in these studies influences systemic inflammation.

As indicated above, the polyamide powder used in this study served as a representative MNP that is not known to be generated with bisphenol analogs or phthalates [53–55] and has no reported endocrine disrupting properties. However, polyamide has a high affinity of estrogenic compounds [80–82] which may act as endocrine disruptors in biological settings and explain the reduction in 17β-estradiol reported in these studies. The consequences of endocrine disruption induced by polyamide inhalation are unknown. Further studies are ongoing to examine how repeated exposure to MNP may affect circulating hormones in females and males, as well as their impact on pregnancy and fetal growth.

The toxicokinetics of MNPs are of equal importance as the toxicodynamics we have addressed in this study. Future studies will aim to visualize MNP in the rat lung as well as translocation to distal tissues after inhalation exposure. Direct interactions of MNPs with biological barriers and other tissues may drive their mobility and toxicity in living systems. Research of other solid particles suggests that their mobility in biological tissues is due, in part, to cellular uptake [83–85], and that they can cause oxidative stress [86, 87], as well as DNA damage [87–89]. Some studies suggest MNPs may have similar outcomes [90], but these parameters have not been investigated.

## Conclusions

In these studies, we present a well characterized rodent whole-body inhalation exposure system that can be used for the toxicological evaluation of MNP inhalation. MNPs are a particle type that has been frequently overlooked despite the likelihood of human exposure and health concerns. To our knowledge, no research group has aerosolized MNP for use in a whole-body exposure chamber. Taken together, our results reveal the immune, cardiovascular, and endocrine systems as potential targets of MNP inhalation after a single exposure. It is imperative that MNPs be investigated for potential toxicity given their ubiquitous nature in the ambient and indoor environment. Furthermore, plastics may act as a toxicological vector due to their ability to adsorb and/or absorb biological, organic, and chemical agents. The pervasive nature of MNPs and their adverse effects as presented in this study highlight the need for better understanding of MNP toxicities to support consumer choice, regulatory policy, and environmental remediation.

## Methods

### Animal model

Female Sprague Dawley rats were purchased from Charles River Laboratories (Kingston, NY) at 8–10 weeks of age. Rats were delivered to an AAALAC accredited vivarium at Rutgers University where they were provided at least 72 h to acclimate. In the vivarium, food and water were available to the rats *ad libitum*. Females were randomly assigned to each group (n = 12 /group) and their estrous cycle status monitored. Estrous cycle stage was confirmed by vaginal smear cytology. Vaginal smears were obtained using cotton swabs moistened with deionized water as previously described [37, 91]. The vaginal mucus and cell populations were transferred to glass slides by rolling the swab on the slides. Using a microscope, the cellular populations in the vaginal orifice of the rat were identified and the stage of the estrous cycle was confirmed. To limit vascular and endocrine variability, all rats were exposed in the estrus phase of their reproductive cycle. All experiments were performed with Rutgers IACUC approval.

### Polyamide powder and bulk characterization

Orgasol® 2001 UD NAT 2, a polyamide ultrafine powder, was obtained from Arkema (King of Prussia, Pennsylvania). Commercial characterization identifies a powder composed of polyamide-12 particles with a nearly round shape and a diameter of 5 ± 1 μm [92]. Physicochemical properties of these particles were confirmed in-house via Brunauer-Emmett-Teller specific surface area analysis. The polyamide powder was characterized as is for SSA using the multi-point BET nitrogen adsorption method in the NOVATouch® LX4 surface area and pore size analyzer (Quantachrome Instruments, Boynton Beach, FL). Using the measured SSA (m^2^/g), an equivalent BET diameter (µm) of the polyamide particles was also computed, assuming a spherical shape and equal size of all particles, using the following equation:

BET Diameter (µm) = 6 / [ρ (g/cm^3^) ´ SSA (m^2^/g)]

where the value for density was 1.14 g/cm^3^ as derived from previous characterization of polyamide [93].

### Inhalation exposure

Rats were exposed to polyamide particles (9.53 ± 1.03 mg/m^3^) or filtered air over an average of 4.35 ± 0.39 or 4.5 h, respectively, using a custom rodent inhalation facility designed for whole-body aerosolized particle inhalation (IEStechno, Morgantown, WV). In brief, particles were aerosolized by an acoustic generator and rats exposed in an 84 L exposure chamber as previously described [43, 94]. The animal inhalation exposure chamber was connected to state-of-the-art real-time and time-integrated instrumentation in order to enable a detailed physicochemical, morphological, and toxicological characterization of the generated and sampled polyamide aerosol. An exposure concentration of near 10 mg/m^3^ was selected based on the American Conference of Governmental Industrial Hygienists (ACGIH) guidelines for poorly soluble and non-cytotoxic inhalable aerosols, as described in the Safety Data Sheet provided by the manufacturer [95]. This concentration is more conservative that the OSHA guidelines of 15 mg/m^3^. Rats were exposed to a concentration comparable to the current average daily concentration assessments of plastic particles in occupational settings [11, 96].

### Real-time and time-integrated particle characterization of polyamide aerosol

The particle number concentrations as a function of size of the generated polyamide aerosol were monitored in real-time using a SMPS (TSI Model 3080, Shoreview, MN) for aerosols in the nano size regime (8–300 nm), and an APS (TSI Model 3321, Shoreview, MN) for submicron- and micron-sized aerosols (0.5–20 μm). The instruments were connected to two separate ports on the exposure chamber via flexible and conductive silicone rubber tubing to minimize particle losses. Measurements were initiated approximately 1 h after the aerosol generation was started to ensure sufficient time for the particle concentrations in the chamber to stabilize. Real-time measurements were collected for at least 1 h. The SMPS recorded a measurement every 2.25 min and the APS recorded every 20 s. The total aerosol number concentrations and the number-size distributions were averaged across the individual measurements over the 1 h monitoring period, and the average aerosol size statistics (i.e. median, mode, mean, geometric mean, and geometric standard deviation) were reported.

The polyamide aerosols were also measured in a size-fractionated manner using a High-Resolution Electrical Low Pressure Impactor (Dekati HR-ELPI+, Kangasala, Finland) or the Harvard Compact Cascade Impactor (CCI) [97]. Four aerodynamic size fractions of the aerosol, i.e., PM_< 0.1_, PM_0.1−2.5_, PM_2.5−10_ and PM_> 10_, were collected in the CCI. The PM_< 0.1_ was collected on Teflon filters (PTFE membrane disc filter: 2 μm pore size, 47 mm diameter, Pall Corporation, Port Washington, NY) while the larger PM were collected on polyurethane foam substrates. The aerosol sampling flow from the exposure chamber was 10 L/min and supplemented with HEPA-filtered ambient air to make up the 30 L/min total flow required by the CCI. Aerosol sampling was continued for 2 h. Post sampling, gravimetric analysis of the PM substrates was performed to calculate the time-averaged aerosol mass concentrations as a function of the different aerodynamic size fractions. Size fractionation measure by the CCI was confirmed with HR-ELPI+.

### Pulmonary deposition modeling of polyamide aerosol

The deposition of the inhaled polyamide aerosol in the different lung regions (i.e. head, tracheobronchial, alveolar) of the exposed virgin female rats was modeled using the Multiple-Path Particle Dosimetry model (MPPD v. 3.04) for a single exposure period of 4 h to 9.53 mg/m^3^ polyamide MNPs, assuming no particle clearance as well as a clearance period of 20 h post exposure [98–100]. The concentration of 9.53 mg/m^3^ was chosen for the model to reflect the average exposure concentration in the experimental animal chamber. The aerosol exposure parameters input into the model were based on real-time monitoring and time-integrated sampling data: Count median diameter = 2.81 μm, GSD = 1.3 and Density of 1.14 g/cm^3^ which has been reported in the literature for polyamide [93]. The following rat lung and breathing parameters were used in the model: Symmetric Sprague Dawley Rat; Body weight = 233 g; Breathing Frequency: 124.6 #/min; TLC = 11.59 mL; FRC = 3.03 mL; Lung (distal) volume = 3.78 mL; URT = 0.348 mL; Tidal volume = 1.6 mL; Inspiratory fraction = 0.5; Pause fraction = 0.0; Body orientation: on stomach; Breathing scenario: Whole-body exposure; Default clearance parameters. The estimated deposited dose of the polyamide particles in the different lung regions was reported in units of particle mass per unit surface area (µg/cm^2^).

### Mean arterial pressure and plasma collection

Twenty-four hr after the exposure, rats were anesthetized using isoflurane (5% induction and 3% maintenance). While anesthetized, animals were weighed, and placed in a nose cone on a surgical board in a supine position where the anesthetic was maintained. Once the animal was secured to the board, the carotid artery was isolated and cannulated with polyethylene tubing with an inner diameter of 0.58 mm and outer diameter of 0.965 mm (BD Intramedic, Franklin Lakes, NJ). MAP was obtained using a BLPR2 pressure transducer as well as a blood pressure monitor (World Precision Instruments, Sarasota, FL). After a stable MAP reading was recorded, approximately 6 mL of blood was collected directly from the cannula into BD Vacutainer® Plus whole blood tubes lined with 100% Dipotassium EDTA Dihydrate. Whole blood was centrifuged (Sorvall™ ST 8, Thermofisher Scientific, Waltham, MA) at 1100 RCF for 10 min (Thermofisher Scientific, Waltham, MA). The plasma was then removed using a pipette, aliquoted for future study, and snap frozen in liquid nitrogen.

### Tissue harvest

The right and left uterine horns were carefully excised with the ovary intact and placed in 4° C physiological salt solution (PSS). The thoracic aorta was also excised and placed in 4° C PSS.

### Pulmonary histology

A subset of animals (18 total) was used for histology and BAL collection. The control rats used for these studies were naïve. Animals housed in the aformentioned AAALAC accredited vivarium breathe filtered air by means of a HEPA filter overlaying the air inlet on their cages. Therefore, the authors cite no reason to assume pulmonary inflammation would differ between naïve and filtered air controls. For all other studies, the stress of the exposure may influence outcome and therefore filtered air controls were used. To our knowledge, cycling reproductive hormones do not affect pulmonary inflammation so estrous cycle was not considered as a factor for histology or BAL analysis.

Lungs were excised and examined for any gross morphological changes. Lungs were perfused with 3% paraformaldehyde in PBS, fixed in 3% paraformaldehyde for 12 h, and then transferred to 50% ethanol. Tissues were embedded in paraffin wax and sectioned. Paraffin-embedded tissues were sectioned at 5 μm, stained with hematoxylin and eosin, and examined by a board-certified veterinary pathologist via light microscopy. Pulmonary histomorphological endpoints for gas exchange and air conduction parenchyma included edema, white blood cell numbers, as well as changes to alveolar walls, alveolar epithelium, and vasculature. Incidence and severity scoring utilized appropriate controls for semiquantitative grades from 0 to 5 with grade 0 indicating none or background changes; grade 1, minimal changes; grade 2, mild changes; grade 3, moderate changes; grade 4, marked changes, and grade 5, severe changes.

### Bronchoalveolar lavage fluid collection and analysis

A 16-gauge blunted needle was inserted in the trachea and secured. Using a syringe, 10 mL of PBS was instilled into the lung and subsequently removed. The collected fluid was centrifuged at 300 RCF for 8 min. The fluid supernatant was collected and stored at -80° until further analysis. Cell pellets were resuspended in 1 mL PBS. BAL cell differentials and albumin content were analyzed as previously described [101, 102].

### Wire myography

Wire myography (DMT, Ann Arbor, MI) was used to assess vessel reactivity of the aorta and the uterine artery. Vessels were excised and placed in cold Wire Myography Physiological Salt Solution (WM-PSS: 130 mM NaCl, 4.7 mM KCl, 1.18 mM KH2PO_4_, 1.17 mM MgSO_4_ 7H_2_O, 14.9 mM NaHCO_3_, 5.5 mM glucose, 0.03 mM EGTA, and 1.6 mM CaCl_2_). Excess connective tissue was removed to isolate the vessel. Segments of the vessel (2 mm) were cut, mounted on two wires within 1 h of tissue harvest, and used for experimentation as previously described [43]. Endothelium-dependent relaxation, endothelium-independent relaxation, and smooth muscle contraction were separately evaluated via cumulative additions of 60 µL of MCh (MP Biomedicals, Solon, OH CAT 190,231), SNP (Thermofisher Scientific, Waltham, MA CAT 211,640,250), and PE (Thermofisher Scientific, Waltham, MA CAT 207,240,100), respectively (10^− 9^ to 10^− 4^ M for each pharmacological application). Responses to chemical agents were randomized.

Wire myography data are presented as a percentage of maximum tension after incubation with High Potassium Physiological Salt Solution (KPSS: 74.7 mM NaCl, 60 mM KCl, 1.18 mM KH_2_PO_4_, 1.17 mM MgSO_4_ 7H_2_O, 1.6 mM CaCl_2_, 14.9 mM NaHCO_3_, 0.03 mM EDTA, and 5.5 mM glucose) for 5 min, which reflects the maximum tension placed on the wire in the myograph chamber by the vessel.

### Pressure myography

The right uterine horn was stabilized in a bath of 4° C Microvessel Physiological Salt Solution (MV-PSS: 119 mM NaCl, 4.7 mM KCl, 1.17 mM MgSO_4_ 7H_2_O, 1.6 mM CaCl_2_ 2 H_2_O, 1.18 mM NaH2PO_4_, 24 mM NaHCO_3_, 5.5 mM glucose, and 0.03 mM EGTA) to visualize the premyometrial radial artery, which is representative of the microcirculation in the uterus [103]. A single radial artery was excised, mounted, and pressurized to 60 mmHg in a bath of MV-PSS prior to experimentation and within 1 h of tissue harvest as described [36, 48, 103]. The equilibrated artery was incubated with various pharmacological agents to measure concentration dependent physiological responses. MCh was used to test endothelium dependent vascular smooth muscle relaxation. SNP was used to test endothelium independent vascular smooth muscle relaxation. PE was used to test smooth muscle contraction. The vessel was exposed stepwise to increasing concentrations (10^− 9^ to 10^− 4^ M) of each given pharmacological agent. The order of execution for each concentration response curve was completely randomized.

Pressure myography data are presented as a percentage of maximum relaxation after incubation with Ca^2+^ Free Microvessel Physiological Salt Solution (Ca^2+^ Free PSS: 120.6 mM NaCl, 4.7 mM KCl, 1.17 mM MgSO_4_ 7H_2_O, 1.18 mM NaH_2_PO_4_, 24 mM NaHCO_3_, 5.5 mM glucose, and 0.03 mM EGTA) for 20 min, which reflects the maximum dilation of the vessel measured by the caliper when maximum dilation was elicited by Ca^2+^ Free PSS.

### Systemic inflammation

Proinflammatory cytokines including IL-6, MCP-1, IL-4, CRP, IFNγ, and IL-5 were measured in plasma using Enzyme-Linked Immunosorbent Assays (ELISAs) (Sigma Aldrich, St. Louis, MO CAT RAB0311, RAB0057 RAB0301, RAB0097 and RAB0227 and Abcam, Cambridge, UK CAT ab267811). For samples with analyte concentrations above the limit of detection but below the level of quantification, a value of half the limit of quantification was assigned as described [104–107].

### Measurement of hormonal mediators of vascular function

ELISA bioassays were used to measure 17β-estradiol (Abcam Cambridge, UK CAT ab108677) and progesterone (Novus Biologicals, Littleton CO CAT NBP2-60127), which are hormonal mediators of vascular function, in plasma. For samples with analyte concentrations above the limit of detection but below the level of quantification, a value of half the limit of quantification was assigned as described [104–107].

### Statistics

Student’s t-test assuming equal variance between groups was used to compare animal characteristics, pulmonary inflammation, and ELISA data. If the data set did not follow a Gaussian distribution and variance was unequal between groups, the Wilcoxon Rank Sum Test was used for comparison. Overall vascular reactivity using pressure myography and wire myography drug response curves were compared using a four-parameter nonlinear regression analysis. GainData (ELISA) analysis tool was used to convert absorbance values to analyte concentrations for each ELISA (Arigo Biolaboratories, Hsinchu City, Republic of China). Statistical analyses were completed with GraphPad Prism 9.0 (San Diego, CA, USA). Data are presented as mean ± SEM and significance is set at p ≤ 0.05.

## Data Availability

The datasets used/analyzed to support the conclusions made in this study are available upon request to the corresponding author.

## Abbreviations

MNP: Micro and nanoplastic particle
PM: Particulate Matter
ENM: Engineered Nanomaterial
MPPD: Multiple-Path Particle Dosimetry
BET: Brunauer–Emmett–Teller
SSA: Specific surface area
SMPS: Scanning Mobility Particle Sizer
APS: Aerodynamic Particle Sizer
CCI: Harvard Compact Cascade Impactor
HR-ELPI: High-Resolution Electrical Low Pressure Impactor
MAP: Mean arterial pressure
BAL: Bronchoalveolar Lavage
MCh: Methacholine
SNP: Sodium nitroprusside
PE: Phenylephrine
PSS: Physiological salt solution
WM-PSS: Wire Myography Physiological Salt Solution
KPSS: High Potassium Physiological Salt Solution
MV-PSS: Microvessel Physiological Salt Solution
Ca2 + Free PSS: Ca2 + Free Microvessel Physiological Salt Solution

## References

[1] Ibrahim YS, Tuan Anuar S, Azmi AA, Wan Mohd Khalik WMA, Lehata S, Hamzah SR (2021). Detection of microplastics in human colectomy specimens. JGH Open.

[2] Ragusa A, Svelato A, Santacroce C, Catalano P, Notarstefano V, Carnevali O (2021). Plasticenta: first evidence of microplastics in human placenta. Environ Int.

[3] Jenner LC, Rotchell JM, Bennett RT, Cowen M, Tentzeris V, Sadofsky LR (2022). Detection of microplastics in human lung tissue using µFTIR spectroscopy. Sci Total Environ.

[4] Leslie HA, van Velzen MJM, Brandsma SH, Vethaak AD, Garcia-Vallejo JJ, Lamoree MH. Discovery and quantification of plastic particle pollution in human blood. Environ Int. 2022:107199. 10.1016/j.envint.2022.107199 35367073

[5] Amato-Lourenço LF, Carvalho-Oliveira R, Júnior GR, Dos Santos Galvão L, Ando RA, Mauad T (2021). Presence of airborne microplastics in human lung tissue. J Hazard Mater.

[6] Ragusa A, Notarstefano V, Svelato A, Belloni A, Gioacchini G, Blondeel C (2022). Raman Microspectroscopy Detection and Characterisation of Microplastics in Human Breastmilk. Polym (Basel).

[7] Advancing Sustainable Materials Management: Facts and Figures Fact Sheet. In: Advancing Sustainable Materials Management: Facts and Figures Report. https://www.epa.gov/sites/default/files/2021-01/documents/2018_ff_fact_sheet_dec_2020_fnl_508.pdf:United States Environmental Protection Agency; 2020.

[8] Levermore JM, Smith TEL, Kelly FJ, Wright SL (2020). Detection of Microplastics in Ambient Particulate Matter using Raman Spectral Imaging and Chemometric Analysis. Anal Chem.

[9] Vianello A, Jensen RL, Liu L, Vollertsen J (2019). Simulating human exposure to indoor airborne microplastics using a Breathing Thermal Manikin. Sci Rep.

[10] Liao Z, Ji X, Ma Y, Lv B, Huang W, Zhu X (2021). Airborne microplastics in indoor and outdoor environments of a coastal city in Eastern China. J Hazard Mater.

[11] Eschenbacher WL, Kreiss K, Lougheed MD, Pransky GS, Day B, Castellan RM (1999). Nylon flock-associated interstitial lung disease. Am J Respir Crit Care Med.

[12] Russ KA, Thompson JA, Kashon M, Porter DW, Friend SA, McKinney W (2019). Comparison of multi-wall carbon nanotube and nitrogen-doped multi-wall carbon nanotube effects on lung function and airway reactivity in rats. Toxicol Appl Pharmacol.

[13] McKinney W, Jackson M, Sager TM, Reynolds JS, Chen BT, Afshari A (2012). Pulmonary and cardiovascular responses of rats to inhalation of a commercial antimicrobial spray containing titanium dioxide nanoparticles. Inhal Toxicol.

[14] Leppänen M, Korpi A, Mikkonen S, Yli-Pirilä P, Lehto M, Pylkkänen L (2015). Inhaled silica-coated TiO2 nanoparticles induced airway irritation, airflow limitation and inflammation in mice. Nanotoxicology.

[15] Tamagawa E, Bai N, Morimoto K, Gray C, Mui T, Yatera K (2008). Particulate matter exposure induces persistent lung inflammation and endothelial dysfunction. Am J Physiol Lung Cell Mol Physiol.

[16] Kido T, Tamagawa E, Bai N, Suda K, Yang HH, Li Y (2011). Particulate matter induces translocation of IL-6 from the lung to the systemic circulation. Am J Respir Cell Mol Biol.

[17] Salvi S, Blomberg A, Rudell B, Kelly F, Sandström T, Holgate ST (1999). Acute inflammatory responses in the airways and peripheral blood after short-term exposure to diesel exhaust in healthy human volunteers. Am J Respir Crit Care Med.

[18] Braakhuis HM, Gosens I, Krystek P, Boere JA, Cassee FR, Fokkens PH (2014). Particle size dependent deposition and pulmonary inflammation after short-term inhalation of silver nanoparticles. Part Fibre Toxicol.

[19] Leung CC, Yu IT, Chen W, Silicosis (2012). Lancet.

[20] Kyjovska ZO, Jacobsen NR, Saber AT, Bengtson S, Jackson P, Wallin H (2015). DNA strand breaks, acute phase response and inflammation following pulmonary exposure by instillation to the diesel exhaust particle NIST1650b in mice. Mutagenesis.

[21] van Eeden SF, Tan WC, Suwa T, Mukae H, Terashima T, Fujii T (2001). Cytokines involved in the systemic inflammatory response induced by exposure to particulate matter air pollutants (PM(10)). Am J Respir Crit Care Med.

[22] Tsai DH, Amyai N, Marques-Vidal P, Wang JL, Riediker M, Mooser V (2012). Effects of particulate matter on inflammatory markers in the general adult population. Part Fibre Toxicol.

[23] Pope CA 3rd, Bhatnagar A, McCracken JP, Abplanalp W, Conklin DJ, O’Toole T. Exposure to fine Particulate Air Pollution is Associated with Endothelial Injury and systemic inflammation. Circ Res. 2016;119 11:1204–14. 10.1161/CIRCRESAHA.116.309279 PMC5215745 27780829

[24] Calderón-Garcidueñas L, Villarreal-Calderon R, Valencia-Salazar G, Henríquez-Roldán C, Gutiérrez-Castrellón P, Torres-Jardón R (2008). Systemic inflammation, endothelial dysfunction, and activation in clinically healthy children exposed to air pollutants. Inhal Toxicol.

[25] Mozzoni P, Iodice S, Persico N, Ferrari L, Pinelli S, Corradi M et al. Maternal air pollution exposure during the first trimester of pregnancy and markers of inflammation and endothelial dysfunction. Environ Res. 2022;212 Pt A:113216. 10.1016/j.envres.2022.113216 35364045

[26] Törnqvist H, Mills NL, Gonzalez M, Miller MR, Robinson SD, Megson IL (2007). Persistent endothelial dysfunction in humans after diesel exhaust inhalation. Am J Respir Crit Care Med.

[27] Liang S, Zhao T, Xu Q, Duan J, Sun Z (2021). Evaluation of fine particulate matter on vascular endothelial function in vivo and in vitro. Ecotoxicol Environ Saf.

[28] Oudin A, Forsberg B, Jakobsson K (2012). Air pollution and stroke. Epidemiology.

[29] Shah AS, Langrish JP, Nair H, McAllister DA, Hunter AL, Donaldson K (2013). Global association of air pollution and heart failure: a systematic review and meta-analysis. Lancet.

[30] Mills NL, Donaldson K, Hadoke PW, Boon NA, MacNee W, Cassee FR (2009). Adverse cardiovascular effects of air pollution. Nat Clin Pract Cardiovasc Med.

[31] Vora R, Zareba W, Utell MJ, Pietropaoli AP, Chalupa D, Little EL (2014). Inhalation of ultrafine carbon particles alters heart rate and heart rate variability in people with type 2 diabetes. Part Fibre Toxicol.

[32] Carll AP, Lust RM, Hazari MS, Perez CM, Krantz QT, King CJ (2013). Diesel exhaust inhalation increases cardiac output, bradyarrhythmias, and parasympathetic tone in aged heart failure-prone rats. Toxicol Sci.

[33] Tobaldini E, Bollati V, Prado M, Fiorelli EM, Pecis M, Bissolotti G (2018). Acute particulate matter affects cardiovascular autonomic modulation and IFN-γ methylation in healthy volunteers. Environ Res.

[34] Rundell KW, Hoffman JR, Caviston R, Bulbulian R, Hollenbach AM (2007). Inhalation of ultrafine and fine particulate matter disrupts systemic vascular function. Inhal Toxicol.

[35] Cuevas AK, Liberda EN, Gillespie PA, Allina J, Chen LC (2010). Inhaled nickel nanoparticles alter vascular reactivity in C57BL/6 mice. Inhal Toxicol.

[36] Stapleton PA, Nichols CE, Yi J, McBride CR, Minarchick VC, Shepherd DL (2015). Microvascular and mitochondrial dysfunction in the female F1 generation after gestational TiO2 nanoparticle exposure. Nanotoxicology.

[37] Stapleton PA, McBride CR, Yi J, Abukabda AB, Nurkiewicz TR (2018). Estrous cycle-dependent modulation of in vivo microvascular dysfunction after nanomaterial inhalation. Reprod Toxicol.

[38] Stefaniak AB, LeBouf RF, Duling MG, Yi J, Abukabda AB, McBride CR (2017). Inhalation exposure to three-dimensional printer emissions stimulates acute hypertension and microvascular dysfunction. Toxicol Appl Pharmacol.

[39] Nurkiewicz TR, Porter DW, Barger M, Castranova V, Boegehold MA (2004). Particulate matter exposure impairs systemic microvascular endothelium-dependent dilation. Environ Health Perspect.

[40] Ain NU, Qamar SUR (2021). Particulate Matter-Induced Cardiovascular Dysfunction: a mechanistic insight. Cardiovasc Toxicol.

[41] Wilson SJ, Miller MR, Newby DE (2018). Effects of Diesel Exhaust on Cardiovascular function and oxidative stress. Antioxid Redox Signal.

[42] Stapleton PA, Minarchick VC, Cumpston AM, McKinney W, Chen BT, Sager TM (2012). Impairment of coronary arteriolar endothelium-dependent dilation after multi-walled carbon nanotube inhalation: a time-course study. Int J Mol Sci.

[43] Fournier SB, Kallontzi S, Fabris L, Love C, Stapleton PA (2019). Effect of gestational age on maternofetal vascular function following single maternal Engineered Nanoparticle exposure. Cardiovasc Toxicol.

[44] Møller P, Christophersen DV, Jacobsen NR, Skovmand A, Gouveia AC, Andersen MH (2016). Atherosclerosis and vasomotor dysfunction in arteries of animals after exposure to combustion-derived particulate matter or nanomaterials. Crit Rev Toxicol.

[45] Stapleton PA, McBride CR, Yi J, Nurkiewicz TR (2015). Uterine microvascular sensitivity to nanomaterial inhalation: an in vivo assessment. Toxicol Appl Pharmacol.

[46] Minarchick VC, Stapleton PA, Porter DW, Wolfarth MG, Çiftyürek E, Barger M (2013). Pulmonary cerium dioxide nanoparticle exposure differentially impairs coronary and mesenteric arteriolar reactivity. Cardiovasc Toxicol.

[47] Gandley RE, Jeyabalan A, Desai K, McGonigal S, Rohland J, DeLoia JA (2010). Cigarette exposure induces changes in maternal vascular function in a pregnant mouse model. Am J Physiol Regul Integr Comp Physiol.

[48] Abukabda AB, Stapleton PA, McBride CR, Yi J, Nurkiewicz TR (2017). Heterogeneous vascular Bed responses to Pulmonary Titanium Dioxide Nanoparticle exposure. Front Cardiovasc Med.

[49] Franklin BA, Brook R, Arden Pope C (2015). 3rd. Air pollution and cardiovascular disease. Curr Probl Cardiol.

[50] Seymore TN, Rivera-Núñez Z, Stapleton PA, Adibi JJ, Barrett ES (2022). Phthalate exposures and placental health in animal models and humans: a systematic review. Toxicol Sci.

[51] Fournier SB, D’Errico JN, Stapleton PA (2021). Uterine vascular control preconception and during pregnancy. Compr Physiol.

[52] Bansal A, Henao-Mejia J, Simmons RA (2018). Immune System: an emerging player in Mediating Effects of Endocrine Disruptors on Metabolic Health. Endocrinology.

[53] Dencheva NV, Braz JFB, Denchev ZZ (2022). Synthesis and properties of neat, hybrid, and copolymeric polyamide 12 microparticles and composites on their basis. J Appl Polym Sci.

[54] Ladkau N, Assmann M, Schrewe M, Julsing MK, Schmid A, Bühler B (2016). Efficient production of the Nylon 12 monomer ω-aminododecanoic acid methyl ester from renewable dodecanoic acid methyl ester with engineered Escherichia coli. Metab Eng.

[55] Turk SCHJ, Kloosterman WP, Ninaber DK, Kolen KPAM, Knutova J, Suir E (2016). Metabolic Engineering toward sustainable production of Nylon-6. ACS Synth Biol.

[56] Oberdörster G, Oberdörster E, Oberdörster J (2005). Nanotoxicology: an emerging discipline evolving from studies of ultrafine particles. Environ Health Perspect.

[57] Kuehl PJ, Anderson TL, Candelaria G, Gershman B, Harlin K, Hesterman JY (2012). Regional particle size dependent deposition of inhaled aerosols in rats and mice. Inhal Toxicol.

[58] Vaughan JM, Garrett BJ, Prophete C, Horton L, Sisco M, Soukup JM (2014). A novel system to generate WTC dust particles for inhalation exposures. J Expo Sci Environ Epidemiol.

[59] Warheit DB, Webb TR, Reed KL, Hansen JF, Kennedy GL (2003). Jr. Four-week inhalation toxicity study in rats with nylon respirable fibers: rapid lung clearance. Toxicology.

[60] Steinle P (2016). Characterization of emissions from a desktop 3D printer and indoor air measurements in office settings. J Occup Environ Hyg.

[61] Bowdridge EC, Abukabda AB, Engles KJ, McBride CR, Batchelor TP, Goldsmith WT (2019). Maternal Engineered Nanomaterial Inhalation during Gestation disrupts vascular kisspeptin reactivity. Toxicol Sci.

[62] Abukabda AB, McBride CR, Batchelor TP, Goldsmith WT, Bowdridge EC, Garner KL (2018). Group II innate lymphoid cells and microvascular dysfunction from pulmonary titanium dioxide nanoparticle exposure. Part Fibre Toxicol.

[63] Vidanapathirana AK, Thompson LC, Herco M, Odom J, Sumner SJ, Fennell TR (2018). Acute intravenous exposure to silver nanoparticles during pregnancy induces particle size and vehicle dependent changes in vascular tissue contractility in Sprague Dawley rats. Reprod Toxicol.

[64] Fuller R, Colton I, Gokina N, Mandala M, Osol G (2011). Local versus systemic influences on uterine vascular reactivity during pregnancy in the single-horn gravid rat. Reprod Sci.

[65] Osol G, Moore LG (2014). Maternal uterine vascular remodeling during pregnancy. Microcirculation.

[66] Orona NS, Astort F, Maglione GA, Ferraro SA, Martin M, Morales C (2020). Hazardous effects of urban air particulate matter acute exposure on lung and extrapulmonary organs in mice. Ecotoxicol Environ Saf.

[67] Marchini T, Magnani ND, Paz ML, Vanasco V, Tasat D, González Maglio DH (2014). Time course of systemic oxidative stress and inflammatory response induced by an acute exposure to residual oil fly Ash. Toxicol Appl Pharmacol.

[68] Cohen MD, Prophete C, Horton L, Sisco M, Park SH, Lee HW (2020). Impact on rats from acute intratracheal inhalation exposures to WTC dusts. Inhal Toxicol.

[69] Uski OJ, Happo MS, Jalava PI, Brunner T, Kelz J, Obernberger I (2012). Acute systemic and lung inflammation in C57Bl/6J mice after intratracheal aspiration of particulate matter from small-scale biomass combustion appliances based on old and modern technologies. Inhal Toxicol.

[70] Gustafsson Ã, Lindstedt E, Elfsmark LS, Bucht A (2011). Lung exposure of titanium dioxide nanoparticles induces innate immune activation and long-lasting lymphocyte response in the Dark Agouti rat. J Immunotoxicol.

[71] Zhang Q, Niu Y, Xia Y, Lei X, Wang W, Huo J (2020). The acute effects of fine particulate matter constituents on circulating inflammatory biomarkers in healthy adults. Sci Total Environ.

[72] Erdely A, Hulderman T, Salmen R, Liston A, Zeidler-Erdely PC, Schwegler-Berry D (2009). Cross-talk between lung and systemic circulation during carbon nanotube respiratory exposure. Potential biomarkers. Nano Lett.

[73] Armstead AL, Minarchick VC, Porter DW, Nurkiewicz TR, Li B (2015). Acute inflammatory responses of nanoparticles in an intra-tracheal instillation rat model. PLoS ONE.

[74] Hedbrant A, Andersson L, Bryngelsson IL, Eklund D, Westberg H, Särndahl E (2020). Quartz dust exposure affects NLRP3 inflammasome activation and plasma levels of IL-18 and IL-1Ra in Iron Foundry Workers. Mediators Inflamm.

[75] Dusza HM, Katrukha EA, Nijmeijer SM, Akhmanova A, Vethaak AD, Walker DI (2022). Uptake, transport, and toxicity of Pristine and Weathered Micro- and nanoplastics in human placenta cells. Environ Health Perspect.

[76] Sinan Subhi Farhan SSM, Saad Abdulrahman Hussain. Effects of progesterone and estradiol on the inflammatory and apoptotic markers of ovariectomized rats challenged with acute septic systemic inflammation. vol. Volume: 9: ssue: 12; 2019.

[77] Straub RH (2007). The complex role of estrogens in inflammation. Endocr Rev.

[78] Villa A, Rizzi N, Vegeto E, Ciana P, Maggi A (2015). Estrogen accelerates the resolution of inflammation in macrophagic cells. Sci Rep.

[79] Butts CL, Shukair SA, Duncan KM, Bowers E, Horn C, Belyavskaya E (2007). Progesterone inhibits mature rat dendritic cells in a receptor-mediated fashion. Int Immunol.

[80] Lara LZ, Bertoldi C, Alves NM, Fernandes AN (2021). Sorption of endocrine disrupting compounds onto polyamide microplastics under different environmental conditions: Behaviour and mechanism. Sci Total Environ.

[81] Han J, Qiu W, Meng S, Gao W (2012). Removal of ethinylestradiol (EE2) from water via adsorption on aliphatic polyamides. Water Res.

[82] Han J, Qiu W, Cao Z, Hu J, Gao W (2013). Adsorption of ethinylestradiol (EE2) on polyamide 612: molecular modeling and effects of water chemistry. Water Res.

[83] Chen W, D’Argenio DZ, Sipos A, Kim KJ, Crandall ED (2021). Biokinetic modeling of nanoparticle interactions with lung alveolar epithelial cells: uptake, intracellular processing, and egress. Am J Physiol Regul Integr Comp Physiol.

[84] Thorley AJ, Ruenraroengsak P, Potter TE, Tetley TD (2014). Critical determinants of uptake and translocation of nanoparticles by the human pulmonary alveolar epithelium. ACS Nano.

[85] Singh S, Shi T, Duffin R, Albrecht C, van Berlo D, Höhr D (2007). Endocytosis, oxidative stress and IL-8 expression in human lung epithelial cells upon treatment with fine and ultrafine TiO2: role of the specific surface area and of surface methylation of the particles. Toxicol Appl Pharmacol.

[86] Møller P, Jacobsen NR, Folkmann JK, Danielsen PH, Mikkelsen L, Hemmingsen JG (2010). Role of oxidative damage in toxicity of particulates. Free Radic Res.

[87] Xie H, Mason MM, Wise JP (2011). Sr. Genotoxicity of metal nanoparticles. Rev Environ Health.

[88] Alarifi S, Ali D, Al-Bishri W (2016). In vitro apoptotic and DNA damaging potential of nanobarium oxide. Int J Nanomedicine.

[89] Wani MR, Shadab G (2020). Titanium dioxide nanoparticle genotoxicity: a review of recent in vivo and in vitro studies. Toxicol Ind Health.

[90] Banerjee A, Shelver WL (2021). Micro- and nanoplastic induced cellular toxicity in mammals: a review. Sci Total Environ.

[91] Goldman JM, Murr AS, Cooper RL (2007). The rodent estrous cycle: characterization of vaginal cytology and its utility in toxicological studies. Birth Defects Res B Dev Reprod Toxicol.

[92] Arkema I. Technical Data Sheet, Orgasol ® NAT2. 2 edn.King of Prussia, Pennsylvania2004.

[93] Lambert S, Wagner M, Wagner M, Lambert S (2018). Microplastics are contaminants of emerging concern in Freshwater environments: an overview. Freshwater Microplastics: emerging environmental contaminants?.

[94] Baron PA, Deye GJ, Chen BT, Schwegler-Berry DE, Shvedova AA, Castranova V (2008). Aerosolization of single-walled carbon nanotubes for an inhalation study. Inhal Toxicol.

[95] Arkema I. Safety Data Sheet, Orgasol® 2001 NAT2. 2.3 edn. King of Prussia, Pennsylvania. 2021.

[96] Antti-Poika M, Nordman H, Nickels J, Keskinen H, Viljanen A (1986). Lung disease after exposure to polyvinyl chloride dust. Thorax.

[97] Demokritou P, Lee SJ, Ferguson ST, Koutrakis P (2004). A compact multistage (cascade) impactor for the characterization of atmospheric aerosols. J Aerosol Sci.

[98] Anjilvel S, Asgharian B (1995). A multiple-path model of particle deposition in the rat lung. Fundam Appl Toxicol.

[99] Miller FJ, Asgharian B, Schroeter JD, Price O (2016). Improvements and additions to the multiple path particle dosimetry model. J Aerosol Sci.

[100] RIVM: Multiple Path Particle Dosimetry Model (MPPD v 1.0) (2002). A model for Human and Rat Airway particle Dosimetry., vol. National Institute for Public Health and the Environment (RIVM). RIVA Report 650010030.

[101] Malaviya R, Venosa A, Hall L, Gow AJ, Sinko PJ, Laskin JD (2012). Attenuation of acute nitrogen mustard-induced lung injury, inflammation and fibrogenesis by a nitric oxide synthase inhibitor. Toxicol Appl Pharmacol.

[102] Sunil VR, Vayas KN, Abramova EV, Rancourt R, Cervelli JA, Malaviya R (2020). Lung injury, oxidative stress and fibrosis in mice following exposure to nitrogen mustard. Toxicol Appl Pharmacol.

[103] Stapleton PA, Minarchick VC, Yi J, Engels K, McBride CR, Nurkiewicz TR (2013). Maternal engineered nanomaterial exposure and fetal microvascular function: does the Barker hypothesis apply?. Am J Obstet Gynecol.

[104] Hornung RW, Reed LD (1990). Estimation of average concentration in the Presence of nondetectable values. Appl Occup Environ Hyg.

[105] Beal SL (2001). Ways to fit a PK model with some data below the quantification limit. J Pharmacokinet Pharmacodyn.

[106] Barnett HY, Geys H, Jacobs T, Jaki T (2021). Methods for non-compartmental pharmacokinetic analysis with observations below the limit of quantification. Stat Biopharm Res.

[107] Keizer RJ, Jansen RS, Rosing H, Thijssen B, Beijnen JH, Schellens JH (2015). Incorporation of concentration data below the limit of quantification in population pharmacokinetic analyses. Pharmacol Res Perspect.

